# Drug Design for CNS Diseases: Polypharmacological Profiling of Compounds Using Cheminformatic, 3D-QSAR and Virtual Screening Methodologies

**DOI:** 10.3389/fnins.2016.00265

**Published:** 2016-06-10

**Authors:** Katarina Nikolic, Lazaros Mavridis, Teodora Djikic, Jelica Vucicevic, Danica Agbaba, Kemal Yelekci, John B. O. Mitchell

**Affiliations:** ^1^Department of Pharmaceutical Chemistry, Faculty of Pharmacy, University of BelgradeBelgrade, Serbia; ^2^School of Biological and Chemical Sciences, Queen Mary University of LondonLondon, UK; ^3^Department of Bioinformatics and Genetics, Faculty of Engineering and Natural Sciences, Kadir Has UniversityIstanbul, Turkey; ^4^EaStCHEM School of Chemistry and Biomedical Sciences Research Complex, University of St AndrewsSt Andrews, UK

**Keywords:** multi-target drugs, CNS disease, QSAR, rational drug design, cheminformatic, virtual screening, virtual docking

## Abstract

**HIGHLIGHTS**

- Many CNS targets are being explored for multi-target drug design
- New databases and cheminformatic methods enable prediction of primary pharmaceutical target and off-targets of compounds
- QSAR, virtual screening and docking methods increase the potential of rational drug design

The diverse cerebral mechanisms implicated in Central Nervous System (CNS) diseases together with the heterogeneous and overlapping nature of phenotypes indicated that multitarget strategies may be appropriate for the improved treatment of complex brain diseases. Understanding how the neurotransmitter systems interact is also important in optimizing therapeutic strategies. Pharmacological intervention on one target will often influence another one, such as the well-established serotonin-dopamine interaction or the dopamine-glutamate interaction. It is now accepted that drug action can involve plural targets and that polypharmacological interaction with multiple targets, to address disease in more subtle and effective ways, is a key concept for development of novel drug candidates against complex CNS diseases. A multi-target therapeutic strategy for Alzheimer‘s disease resulted in the development of very effective Multi-Target Designed Ligands (MTDL) that act on both the cholinergic and monoaminergic systems, and also retard the progression of neurodegeneration by inhibiting amyloid aggregation. Many compounds already in databases have been investigated as ligands for multiple targets in drug-discovery programs. A probabilistic method, the Parzen-Rosenblatt Window approach, was used to build a “predictor” model using data collected from the ChEMBL database. The model can be used to predict both the primary pharmaceutical target and off-targets of a compound based on its structure. Several multi-target ligands were selected for further study, as compounds with possible additional beneficial pharmacological activities. Based on all these findings, it is concluded that multipotent ligands targeting AChE/MAO-A/MAO-B and also D_1_-R/D_2_-R/5-HT_2*A*_-R/H_3_-R are promising novel drug candidates with improved efficacy and beneficial neuroleptic and procognitive activities in treatment of Alzheimer's and related neurodegenerative diseases. Structural information for drug targets permits docking and virtual screening and exploration of the molecular determinants of binding, hence facilitating the design of multi-targeted drugs. The crystal structures and models of enzymes of the monoaminergic and cholinergic systems have been used to investigate the structural origins of target selectivity and to identify molecular determinants, in order to design MTDLs.

## Polypharmacology of compounds against CNS diseases

Traditional drug discovery methods have mainly been based on development of selective agents for a specific target able to modulate its activity and the pathophysiology of the disease. This approach in now generally recognized as too simplistic for designing effective drugs to address complex multifactorial diseases, characterized by diverse physiological dysfunctions caused by dysregulation of complex networks of proteins (Anighoro et al., 2014). Modern drug design of multitarget ligands able to specifically modulate a network of interacting targets and show unique polypharmacological profiles is becoming increasingly important in drug discovery for multifactorial pathologies such as complex central nervous system (CNS) diseases (Hopkins, 2008; Mestres and Gregori-PuigjaneÌĄ, 2009; Boran and Iyengar, 2010; Peters, 2013; Anighoro et al., 2014).

The most significant advantages of the use of multitarget drugs over other therapeutic strategies, such as polypharmaceutical or single-targeted therapy, are: improved efficacy as result of synergistic or additive effects caused by simultaneous and specific interactions with chosen palette of biological targets; better distribution in target tissue for simultaneous action on multiple targets; accelerated therapeutic efficacy in terms of initial onset and achievement of full effect; treatment of broader therapeutic range of symptoms; predictable pharmacokinetic profile and mitigated drug-drug interactions; lower incidence of molecule-based side effects; increased therapeutic interval of doses as result of lower risk of acute and delayed toxicity; better quality of treatment; improved patient compliance and tolerance; and lower incidence of target-based resistance as result of modulation of multiple targets (Millan, 2006, 2014; Anighoro et al., 2014). The main challenge in drug discovery of MTDLs is to develop an efficient methodology for the design of novel multipotent drugs able to interact only with one additional target and without significant affinities for other related targets.

The polypharmacological design of CNS drugs is challenging because of the complex pathophysiological mechanisms of brain diseases, interactions of neurotransmitter systems and observed ligand cross-reactivities (Roth et al., 2004). Since multipotent ligands could also interact with off-targets and cause target-based adverse effects, a major objective in polypharmacology is to rationally design multi-target drugs able to specifically modulate only a group of desired targets while minimizing interactions with off-targets and avoiding interactions with anti-targets (Anighoro et al., 2014; Millan, 2014). Multi-Target Designed Ligands (MTDL) contain the primary pharmacophore elements for each target which could be separated by a linker (conjugate MTDLs), could touch at one point (fused), or could be combined by using commonalities in the structures of underlying pharmacophores (merged) (Besnard et al., 2012; Millan, 2014).

Smaller and relatively rigid structures of highly merged MTDLs result in better physicochemical, pharmacokinetic and pharmacological profiles (Besnard et al., 2012; Millan, 2014). For the rationally designed MTDLs, activities against the targets and pharmacokinetic profiles are predicted. Based on the results obtained, the most promising MTDLs are selected for further modifications and studies (Hajjo et al., 2010; Besnard et al., 2012; Hajjo et al., 2012; Zhang et al., 2013; Nikolic et al., 2015a).

Several previous studies confirmed that multifactorial pathologies, such as cerebral mechanisms implicated in neurological and psychiatric diseases (Threlfell et al., 2004; Dai et al., 2007; Garduno-Torres et al., 2007; Humbert-Claude et al., 2007; Gemkow et al., 2009) and neurodegenerative disorders (Goedert and Spillantini, 2006), are often polygenic and involve the dysregulation of very complex networks of proteins. The diverse cerebral mechanisms implicated in CNS diseases together with the heterogeneous and overlapping nature of phenotypes indicated that multitarget strategies may be appropriate for improved treatment of complex brain diseases. Both the activity and the side effects of CNS drugs are characterized by a complex pattern of biological activities on multiple targets and a complex mechanism of action (Roth et al., 2004; Lipina et al., 2012, 2013). Understanding how the neurotransmitter systems interact is also important in optimizing therapeutic strategies. Pharmacological intervention on the dopamine system will often influence the serotonin or glutamate neurotransmitter systems. Interactions of the neurotransmitter systems, such as the dopamine-glutamate interaction (Carlsson and Carlsson, 1990; Millan, 2005) and the serotonin-dopamine interaction (Di Giovanni et al., 2008; Di Matteo et al., 2008), are also very important factors in design of multitargeted ligands with specific cross-reactivity and optimized neuropharmacological effects (Youdim and Buccafusco, 2005). Therefore, a more efficient polypharmacological strategy for treatment of complex CNS diseases is based on drug interactions with multiple targets, to address disease in more subtle and effective ways while avoiding side effects arising from interaction with defined antitargets and off-targets (Lu et al., 2012; Anighoro et al., 2014). Thus, polypharmacology is now recognized as a key pharmacological concept for development of novel drug candidates against complex CNS diseases.

As a result of the multitarget approach (Morphy and Rankovic, 2005; León et al., 2013; Anighoro et al., 2014; Millan, 2014) many CNS drugs with improved efficacy compared to their lead compounds have been developed and examined. Monoamine reuptake inhibitors with serotonin 5-HT_2C_ antagonistic properties were developed as novel class of antidepressants (Millan, 2006; Meltzer et al., 2012; Quesseveur et al., 2012). Dopamine receptors are G protein–coupled receptors (GPCRs), distinct in pharmacology, amino acid sequence, distribution, and physiological function. Based on their effector-coupling profiles dopamine receptors are organized into two families, the D_1_-like (D_1_, D_5_) and D_2_-like (D_2_, D_3_, D_4_) receptors (Brunton et al., 2011).

The physiological processes under dopaminergic control include reward, emotion, cognition, memory, and motor activity. Therefore, dysregulation of the dopaminergic system is critical in a number of disease states, including Parkinson's disease, Tourette's syndrome, bipolar depression, schizophrenia, attention deficit hyperactivity disorder, and addiction/substance abuse (Brunton et al., 2011). Dopamine receptor antagonists are a mainstay in the pharmacotherapy of schizophrenia.

Since the pathophysiology of schizophrenia and related diseases involves deregulation of the dopamine, serotonin and glutamate neurotransmitter systems (Witkin and Nelson, 2004; Esbenshade et al., 2008; Brunton et al., 2011), therapeutic effects of typical and atypical neuroleptics are mostly mediated by inhibition of dopamine D_1_/D_2_-like receptors and other related aminergic receptors (Table 1). Blockade of dopamine D_2_ and serotonin 5-HT_2A_ receptors is the main mechanism of action of atypical antipsychotics (Remington, 2003). Furthermore, interaction with various dopamine (D_1_, D_3_, D_4_), serotonin (5-HT_1A_, 5-HT_1D_, 5-HT_2A_, 5-HT_2C_, 5-HT_6_, and 5-HT_7_), and histamine H_3_ receptors may produce additional antipsychotic or procognitive effects (Reynolds, 2004; Esbenshade et al., 2008; Coburg et al., 2009) by indirectly modulating the mesolimbic dopaminergic neurons (Amato, 2015).

**Table 1:** **Polypharmacological profiles of drugs and drug candidates affecting the dopaminergic system**.

**Compound**	**Targets**
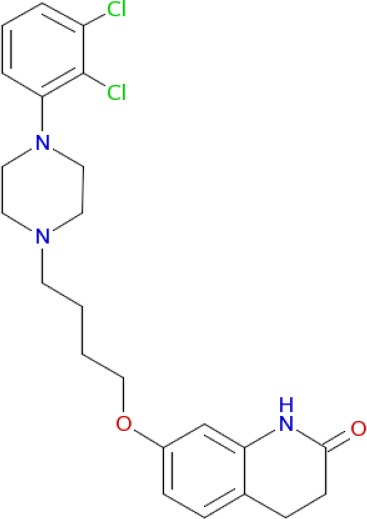 Aripiprazole (Johnson et al., 2011)	D_2_, D_3_, 5-HT_2B_, D_4_, 5-HT_2A_, 5-HT_1A_, 5-HT_7_, α_1A_, H_1_ receptors (Buckley, 2003; Shapiro et al., 2003)
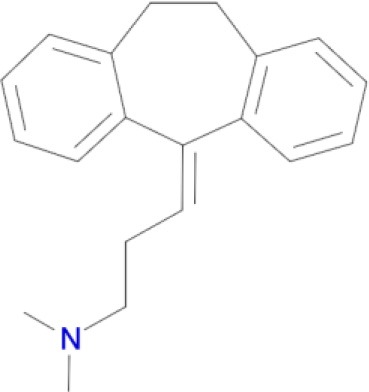 Amitriptyline (Coburg et al., 2009)	D_1_, D_5_, D_2_, D_3_, H_1_ receptors (Ligneau et al., 2000)
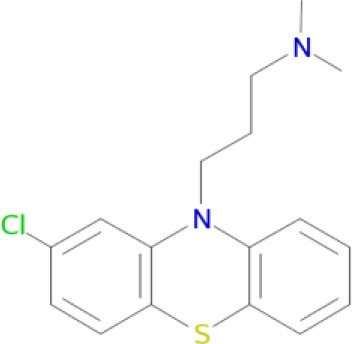 Chlorpromazine (Bourne, 2001)	D_1_, D_5_, D_2_, D_3_, D_4_, 5-HT_2a_ receptors (Rajagopalan et al., 2014)
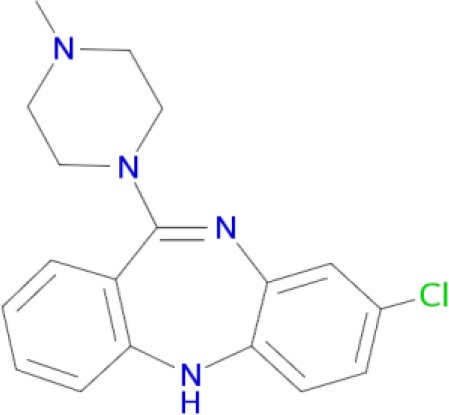 Clozapine (Coburg et al., 2009)	D_1_, D_5_, D_2_, D_3_, D_4_, 5-HT_2A_, H_1_ receptors (Ligneau et al., 2000; Bourne, 2001; Rajagopalan et al., 2014)
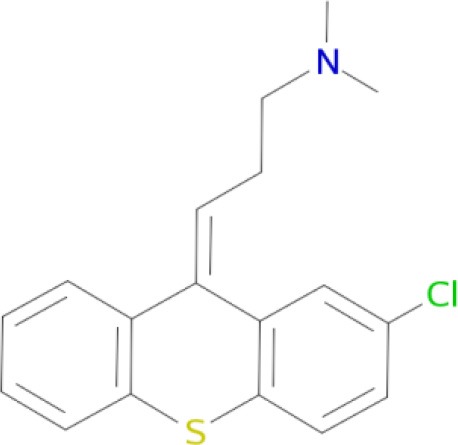 Chlorprothixene (Coburg et al., 2009)	D_1_, D_5_, D_2_, D_3_, D_4_, H_1_ receptors (Ligneau et al., 2000)
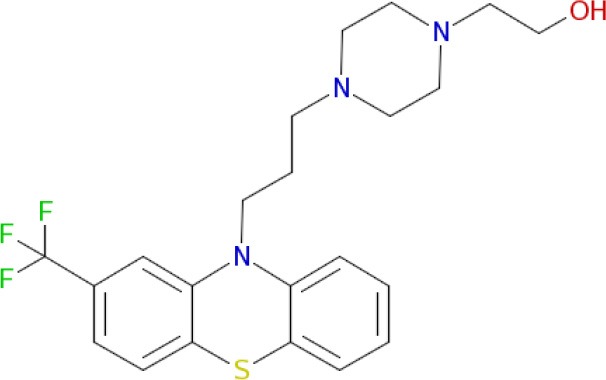 Fluphenazine (Coburg et al., 2009)	D_1_, D_5_, D_2_, D_3_, D_4_, H_1_ receptors (Ligneau et al., 2000)
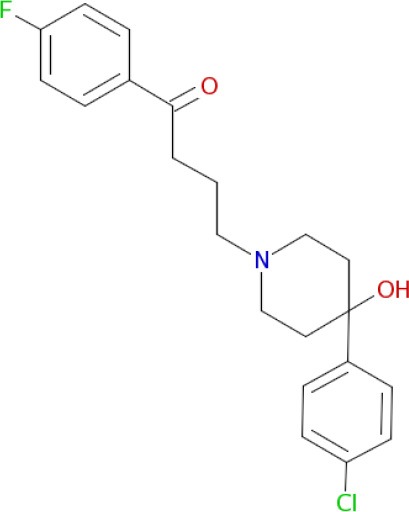 Haloperidol (Bourne, 2001)	D_1_, D_5_, D_2_, D_3_, D_4_, 5-HT_2A_ receptors (Hamacher et al., 2006)
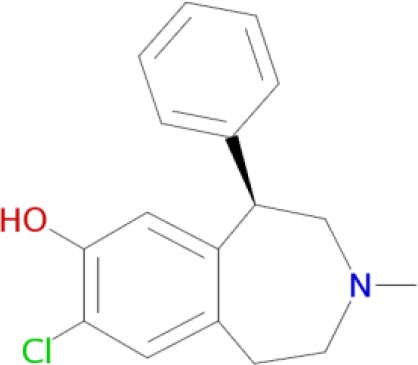 SCH 23390 (Bourne, 2001)	D_1_, D_5_, D_2_, D_3_, D_4_, 5-HT_2A_, 5-HT, α_2A_receptors (Wu et al., 2005)
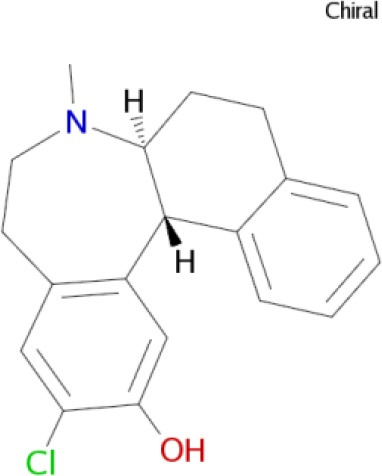 SCH 39166 (Wu et al., 2005)	D_1_, D_5_, D_2_, 5-HT, α_2A_ receptors
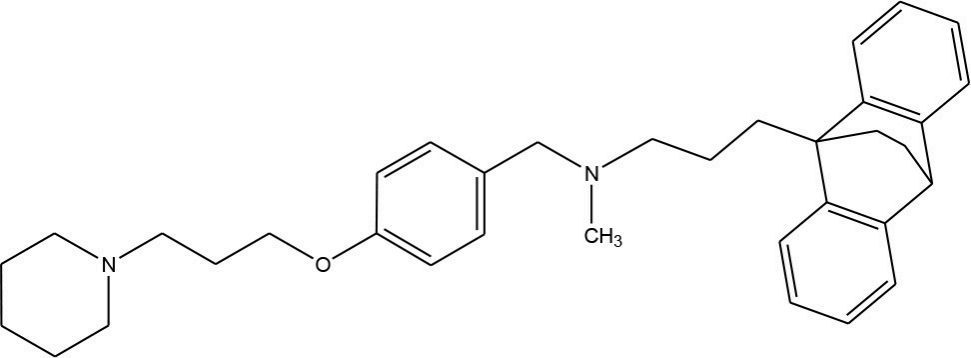 13 (Coburg et al., 2009)	D_1_, D_5_, D_2_, D_3_, D_4_, H_1_, H_3_ receptors (Ligneau et al., 2000; Bourne, 2001; Hamacher et al., 2006; Rajagopalan et al., 2014)

A significant improvement in schizophrenia therapy came in the early 2000s with the use of aripiprazole acting as a dopamine D_2_-like partial agonist with partial agonistic properties on serotonergic 5-HT_1A_ and 5-HT_2A_ receptors (Buckley, 2003; Kiss et al., 2010; Johnson et al., 2011). Dopamine D_2_/D_3_ antagonists, with 5-HT_2A_ antagonistic and 5-HT_1A_ partial agonistic activities, were proposed as drug candidates for schizophrenia therapy (Roth et al., 2004; Lipina et al., 2012, 2013). The efficient polypharmacological profile of aripiprazole and related antipsychotics resulted in the development of cariprazine and pardoprunox as drug candidates, which are currently in clinical trials (Ye et al., 2014).

Despite selective D_1_ antagonism not being accepted on its own as an effective antipsychotic principle (Table 1; Tauscher et al., 2004; Sedvall and Karlsson, 2006), moderate antagonistic activity at D_1_-receptors has been confirmed to be responsible for atypical neuroleptic clozapine effectiveness against treatment-resistant schizophrenia (Tauscher et al., 2004). Based on the polypharmacological profiles of recently approved antipsychotic drugs, it could be concluded that optimal and balanced modulation of D_1_/D_2_-like receptors - as well as interaction with serotonin and histamine H_3_ receptors - should provide the most favorable neuroleptic effect. The successfully developed effective MTDLs with optimal polypharmacological profile for CNS diseases (Table 1) are experimental proof of the polypharmacological concept. Polypharmacological approaches are therefore likely to be extensively applied for rational design of ligands with optimal multitarget profile and for discovery of multipotent drug candidates with improved efficacy and safety in therapy of complex brain diseases.

Novel procognitive agents were developed as histamine H_3_R antagonists/inverse agonists with inhibition of acetylcholine esterase (AChE), monoamine oxidase (MAO), histamine N-methyltransferase (HMT), or serotonin transporter (SERT) (Ligneau et al., 1998; Apelt et al., 2002, 2005; Grassmann et al., 2003, 2004; Petroianu et al., 2006; Decker, 2007; Esbenshade et al., 2008; Sander et al., 2008; Coburg et al., 2009; Bajda et al., 2011; Nikolic et al., 2015a). Rasagiline and ladostigil, drugs currently used as selective MAO-B inhibitors in therapy of PD, contain the propargylamine scaffold and therefore exert significant neuroprotective activity. Thus, phase II clinical trials of rasagiline (www.clinicaltrials.gov/ct2/show/NCT00104273) and ladostidil (www.clinicaltrials.gov/ct2/show/NCT01354691) in therapy of AD were proposed, and subsequently successfully completed. A multi-target therapeutic strategy for Alzheimer‘s disease resulted in the development of very effective MTDLs that act on both the cholinergic and monoaminergic systems, and also retard the neurodegenerative progress by inhibiting amyloid aggregation. Multi-target inhibitors of acetylcholine esterase and MAO (AChE/BuChE/MAO-A/MAO-B) were effective drug candidates for therapy of neurodegenerative Alzheimer's (AD) and Parkinson's diseases (PD) (Pérez et al., 1999; Marco-Contelles et al., 2006, 2009; Bolea et al., 2011; León et al., 2013; Bautista-Aguilera et al., 2014a,c; Nikolic et al., 2015b).

Besides the difficulties of effective modulation of the CNS targets, the need to design drugs that are able to reach the targets in the brain increases the complexity of CNS drug discovery. This is mainly due to the blood-brain barrier (BBB) protection system between the blood capillaries of the brain and brain tissue (Pardridge, 2005). The BBB enables selective access of required nutrients and hormones, while removing waste and preventing or reducing penetration of xenobiotics (Pardridge, 2005). Therefore, a major challenge in CNS drug discovery is to build and apply relationships between chemical structure and brain exposure (Rankovic and Bingham, 2013; Rankovic, 2015a). Total brain concentration (Cb) is now recognized as being only a portion of the non-specific binding to brain tissue, while the unbound brain concentration (Cu,b) is defined as the drug concentration at the target sites and is a measure of *in vivo* drug efficacy. Finally, receptor occupancy (RO) is direct measure of target engagement (Rankovic, 2015b). Lipophilicity of CNS drugs is generally considered the most critical physicochemical parameter for improved penetration and potency. Higher lipophilicity causes low solubility, high plasma protein binding, and increased metabolic and toxicity risks in CNS drugs (Leeson and Springthorpe, 2007). Furthermore, hydrogen bond molecular parameters are the dominant descriptors for unbound drug brain concentrations (Leeson and Davis, 2004). Reducing the HBD (Hydrogen Bond Donor) count of a molecule is one of the most successful strategies used in the optimization of brain exposure (Weiss et al., 2012). In CNS drug discovery, aqueous solubility is also considered in combination with the previously described parameters. Most of the CNS drugs with low safety risk are very soluble compounds, displaying aqueous solubility of more than 100 μM (Alelyunas et al., 2010). Generally, fine-tuning physicochemical properties for optimal brain exposure is now an essential method in CNS drug discovery (Table 2). Further studies of CNS property space and development of predictive models for brain exposure should result in the formation of a general methodology with a wide applicability domain in CNS drug design.

**Table 2:** **Developing CNS property space for optimal brain exposure (Rankovic and Bingham, 2013; Rankovic, 2015b)**.

**CNS property space**
TPSA < 60 Å^2^, p*K*a < 8 and HBD count < 2 are minimizing P-gp recognition (Hitchcock, 2012; Desai et al., 2013)
TPSA (25–60 Å^2^); at least one N atom; linear chains outside of rings (2–4); HBD (0–3); volume (740–970 Å^3^); SAS (460–580 Å^2^) → ↑BBB penetration (Ghose et al., 2012)
Optimal cLogP < 3 (Gleeson, 2008)
cLogP < 4 and TPSA 40–80 Å^2^ → ↑*C*u,b (Raub et al., 2006)
PSA < 90 Å^2^; HBD < 3; cLogP 2–5; cLogD (pH 7.4) 2–5; and MW < 500 → ↑BBB penetration (Hitchcock and Pennington, 2006)
MW < 450; cLogP < 5; HBD < 3; HBA < 7; RB < 8; H-bonds < 8; p*K*a 7.5–10.5; PSA < 60–70 Å^2^. → ↑BBB penetration (Pajouhesh and Lenz, 2005)

TPSA, topological polar surface area; Å^2^, square angstrom; Å^3^, qubic angstrom; HBD, hydrogen-bond donors; P-gp, P-glycoprotein; BBB, blood-brain bariere; HBA, hydrogen-bond acceptors; MW, molecular weight; PSA, polar surface area; cLogP, partition coefficient; cLogD, distribution coefficient; RB, rotatable bonds; Cu,b, unbound drug concentrations in brain; ↓, decreased; ↑, increased.

## 3D-QSAR study of multitarget compounds for CNS diseases

QSAR (*Quantitative Structure-Activity Relationship*) modeling has progressed from analysis of small series of congeners using basic regressions to applications on very large and diverse data sets using a variety of statistical and machine learning methods. Today's QSAR practice widely uses ligand based theoretical approaches for modeling the physical, biological and pharmacological properties of compounds, and forms a crucial initial step in drug discovery. Together with structure-based methods, statistically based QSAR techniques are essential tools in lead optimization within several leading drug discovery groups (Cramer, 2012; Cherkasov et al., 2014).

Modern QSAR methodologies started with a 1962 publication by the Hansch group (Hansch et al., 1962), and further developed with the exploration of series of congeners (Craig, 1971; Topliss, 1972; Hansch et al., 1973). Steric effects of substituents were successfully described by five shape descriptors for substituents (Verloop et al., 1976). Electrostatic interaction energies in a series of superimposed 3D-conformations of analogs were effectively included in CoMFA (*Comparative Molecular Field Analysis*) and other 3D-QSAR methods (Cramer et al., 1988). In CoMFA, steric and electrostatic molecular fields of ligands are calculated and correlated with bioactivities by use of PLS (*Partial Least Squares*) (Wold et al., 1984). Based on the CoMFA approach, the CoMSIA method (*Molecular Similarity Indices in a Comparative Analysis*) was developed (Klebe et al., 1994), encompassing the steric, electrostatic, hydrogen bonding and hydrophobic effects of ligands. The main limitation of CoMFA/CoMSIA and other 3D-QSAR methods relates to their being applicable only to static structures of chemical analogs, while neglecting the dynamical nature of the ligands (Acharya et al., 2011).

Molecular field generating software, such as GRID (Goodford, 1985) and PHASE (Dixon et al., 2006), historically applied pharmacophoric constraints to facilitate 3D-QSAR modeling, considering multiple conformations. The new generation of 3D-descriptors, such as GRIND/GRIND-2/GRID-PP (*Grid-Independent Descriptor*), are alignment free descriptors derived from the *Molecular Interaction Fields* (MIF) of the series and designed to retain the chemical characteristics of the ligands examined. The GRIND descriptors so obtained are provided by programs from Molecular Discovery (Pastor et al., 2000; Durán et al., 2009) and used for advanced multivariate analyses and 3D-QSAR modeling.

**Figure 1:**
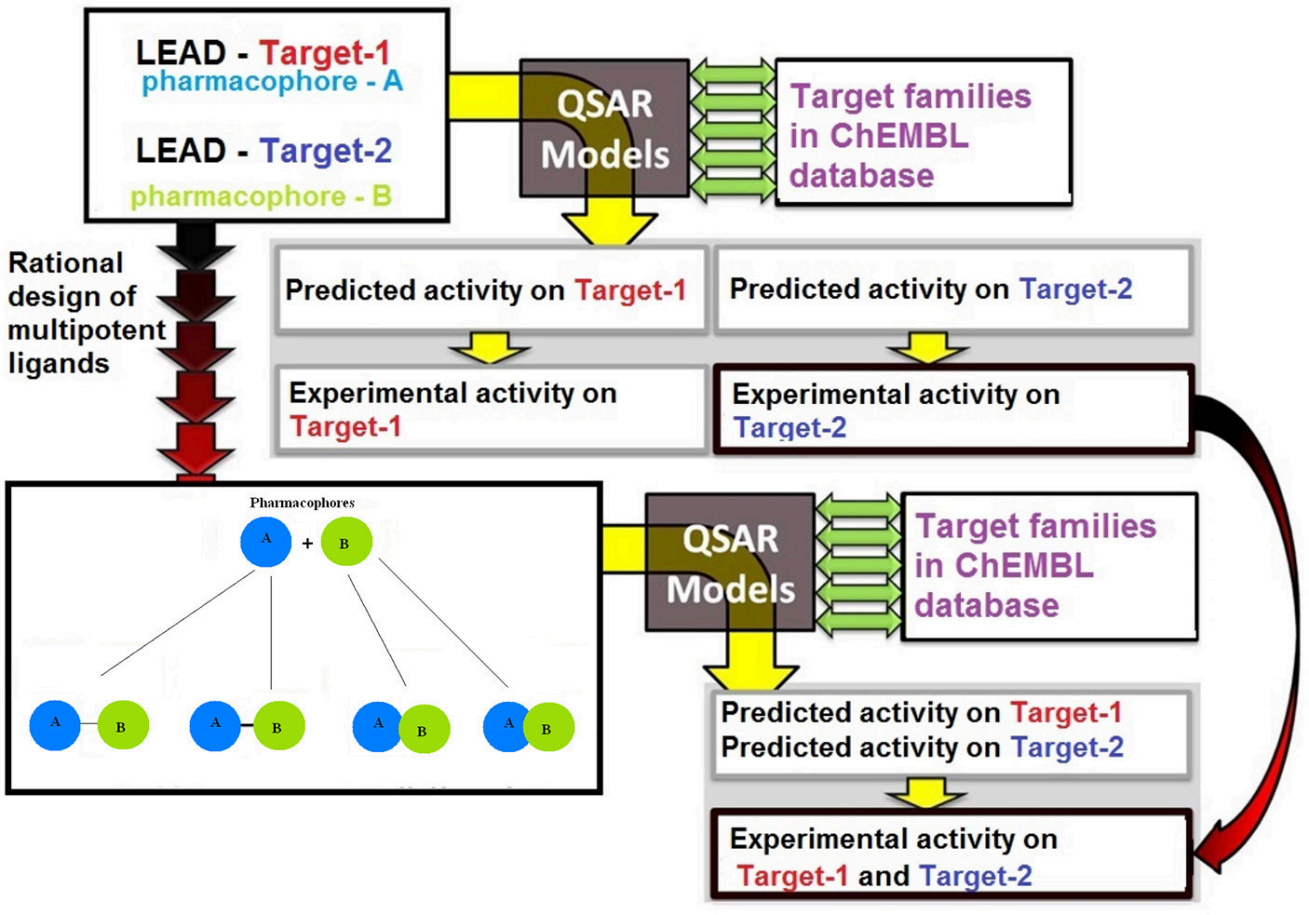
**Computer-aided rational design of multipotent ligands with controlled polypharmacology**.

Some novel 3D-QSAR approaches based on ligand-based 3D-QSAR models and complementary drug target fields are included in the AFMoC (Gohlke and Klebe, 2002) and QMOD (Varela et al., 2012) programs. The QSAR study of multitarget compounds involves QSAR modeling for each target activity individually, study of all developed QSAR models as part in a network of interrelated models, and design of novel multipotent compounds (Cherkasov et al., 2014). Combinations of the QSAR approach and related theoretical methods, such as virtual screening and docking, are very useful in the study and design of multitarget ligands with unique polypharmacological profiles (Figure 1; Ning et al., 2009; Zheng et al., 2010; Besnard et al., 2012; Kupershmidt et al., 2012; Bolea et al., 2013; Bautista-Aguilera et al., 2014c). Based on the developed QSAR models, analogs of a multitarget lead are designed with enhanced activity on the targets and optimal polypharmacological and safety profiles as drug candidates for further study. Recently developed QSAR approaches were the only *in silico* methodologyies able to distinguish between antagonists and agonists of olfactory receptors (ORs), a superfamily of G-protein coupled receptors (Don and Riniker, 2014).

Several successful cases of reported 3D-QSAR studies used in CNS drugs discovery have been listed in Table 3. In this chapter we provide an overview of some of them. For example, polypharmacological profiles of *in silico* generated analogs of donepezil, an approved acetylcholinesterase inhibitor drug, were evaluated by a QSAR study. More than 75% of the ligand-target predictions were confirmed by *in vitro* testing (Besnard et al., 2012). Pathophysiology of Alzheimer's disease (AD) includes extracellular deposition of amyloid β peptide (Aβ)-containing plaques, progressive loss of cholinergic neurons, metal dyshomeostasis, mitochondrial dysfunction, neuroinflammation, oxidative stress and increased MAO enzyme activity. Furthermore, levels of neurotransmitters such as dopamine, noradrenaline, and serotonin are significantly decreased in AD patients (Reinikainen et al., 1990). MAO-A/B inhibitors could increase the levels of dopamine, noradrenaline, and serotonin in the CNS. Therefore, MAO-A/B inhibitors have also been proposed as potential drugs for AD (Youdim et al., 2006).

**Table 3:** **Reported 3D-QSAR studies used in CNS drug discovery**.

**Drug target**	**CNS disease**	**3D-QSAR method**	**Software package**	**References**
MAO-A, MAO-B, AChE, BuChE	AD	GRID based 3D-QSAR modeling (Goodford, 1985; Pastor et al., 2000; Durán et al., 2009)	Pentacle www.moldiscovery.com	Bautista-Aguilera et al., 2014a,b
AChE	AD	Molecular field based 3D-QSAR modeling (Dixon et al., 2006)	PHASE www.schrodinger.com	Lakshmi et al., 2013
AChE, BuChE	AD	CoMFA based 3D-QSAR modeling Wold et al. (1984)	Tripos Sybyl www.tripos.com	Li et al., 2013
AChE	AD	3D multi-target QSAR (Prado-Prado et al., 2012)	DRAGON http://www.talete.mi.it/ MARCH-INSIDE (MARkovian CHemicals IN SIlico DEsign)	González-Díaz et al., 2012
H_3_-R, HMT, AChE, BuChE	AD, PD, depression, schizophrenia	Molecular field and GRID based 3D-QSAR modeling (Goodford, 1985; Pastor et al., 2000; Dixon et al., 2006; Durán et al., 2009)	PHASE www.schrodinger.com Pentacle www.moldiscovery.com	Nikolic et al., 2015a

**Figure 2:**
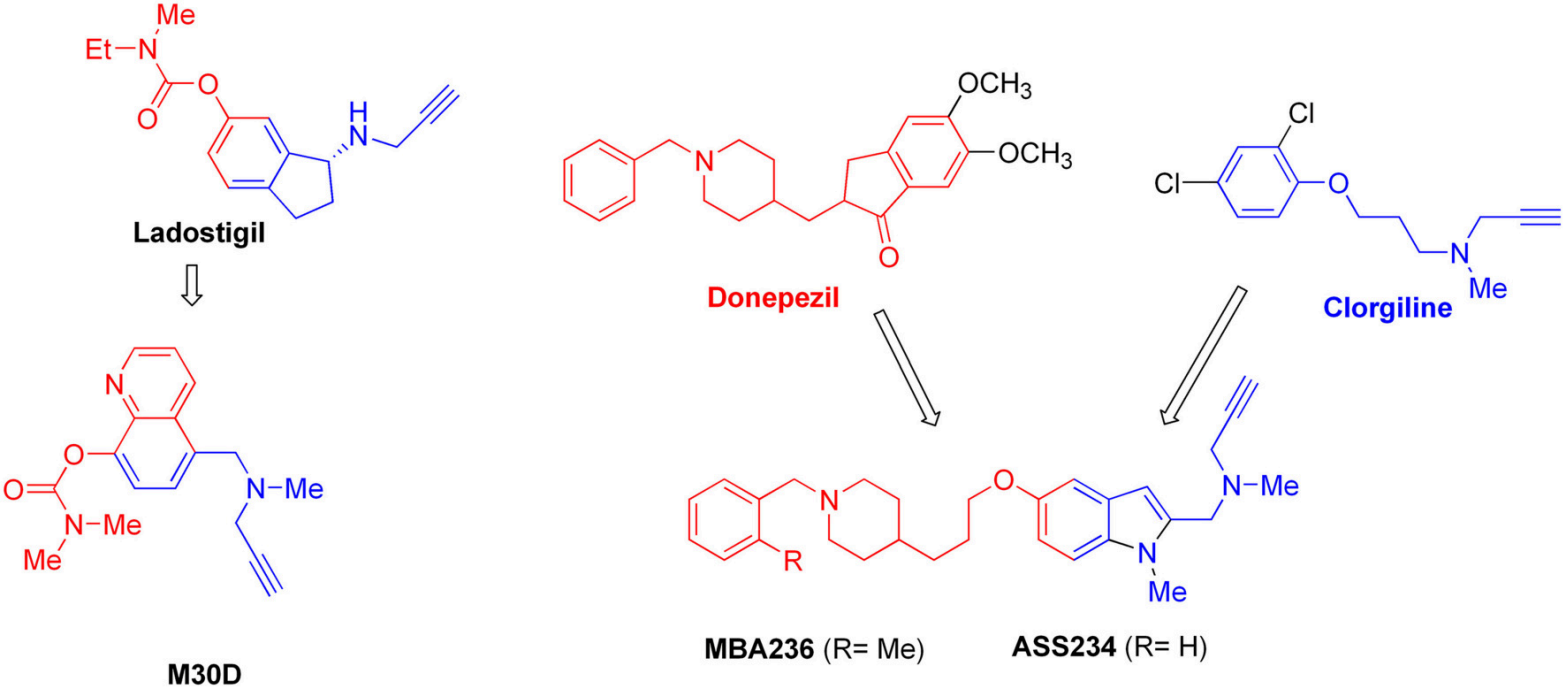
**Structures and pharmacophores of effective Multi-Target Designed Ligands against AD**. Blue coloring represents the MAO inhibitor pharmacophore and red represents the ChE inhibitor pharmacophore.

Multimodal brain permeable drugs affecting a few brain targets involved in the disease pathology, such as MAO and ChE enzymes, iron accumulation and amyloid-β generation/aggregation, were extensively examined as an essential therapeutic approach in AD treatment (Zheng et al., 2010; Bautista-Aguilera et al., 2014b). For instance, hybrid compound **M30D** contains the important pharmacophores from three drugs: tacrine, rivastigmine (ChEIs) and rasagiline/ladostigil (MAO-B inhibitor) (Zheng et al., 2010), while **ASS234** and **MBA236** contain the pharmacophores of the drugs donepezil (ChEIs) and clorgiline (MAO-A inhibitor) (Bolea et al., 2011). Pharmacophore and 3D-QSAR studies of donepezil and clorgiline derivatives inhibiting both AChE/BuChE and MAO-A/B were successfully applied for lead optimization work and for design of **ASS234**, **MBA236** and related ligands with optimal polypharmacological and pharmacokinetic profiles (Bautista-Aguilera et al., 2014a,b,c). The propargylamine moiety in the MAO-inhibiting pharmacophore of rasagiline, ladostigil or clorgiline is responsible for their neuroprotective-neurorestorative effects. Therefore, the propargylamine moiety was used as the main chemical scaffold responsible for MAO inhibition in the designed **M30D**, **ASS234**, and **MBA236** hybrids (Figure 2). Hybrid compound **ASS234** acted as an 11-fold less potent MAO-A inhibitor and 54-fold more potent MAO-B inhibitor than the reference compound clorgiline, while **MBA236** was nine times more potent as an MAO-A inhibitor and 6-fold more potent for MAO-B than reference compound **ASS234**. Inhibition of the ChEs by the hybrid **MBA236** is in the micromolar range, slightly better than compound **ASS234** for AChEs while slightly poorer for BuChE (Table 4; Bautista-Aguilera et al., 2014b). The Multi-Target Designed Ligand **M30D** was found to be a highly potent inhibitor of MAO-A with moderate MAO-B inhibiting activity. Also, **M30D** was a more potent AChE inhibitor than rivastigmine, while rivastigmine was a much stronger BuChE inhibitor than **M30D** (Table 4; Zheng et al., 2010). Further to their MAO/ChE inhibitory properties, **ASS234** and **M30D** exert beneficial pharmacological effects in therapy of AD by inhibiting Aβ plaque formation and aggregation and, by blocking AChE-mediated Aβ1-40/Aβ1-42 aggregation (Kupershmidt et al., 2012; Bolea et al., 2013).

**Table 4:** **IC50 values for the inhibitory effects of test compounds on the enzymatic activity of MAO-A, MAO-B, AChE, and BuChE**.

**Compound**	**MAO-A**	**MAO-B**	**AChE**	**BuChE**
MBA236^a^	6.3 ± 0.4 nM	183.6 ± 7.4 nM	2.8 ± 0.1 μM	4.9 ± 0.2 μM
ASS234^a^	58.2 ± 1.2 nM	1.2 ± 0.1 μM	3.4 ± 0.2 μM	3.3 ± 0.2 μM
Clorgiline^a^	4.7 ± 0.2 nM	65.8 ± 1.6 μM	^**^	^**^
M30D^b^	7.7 ± 0.7 nM	7.9 ± 1.3 μM	0.5 ± 0.1 μM	44.9 ± 6.1 μM

a Bautista-Aguilera et al. (2014b).

b Zheng et al. (2010).

** Inactive at 100 μM (highest concentration tested).

## Cheminformatics methods for on-target and off-target bioactivity prediction

The prediction of interactions between druglike organic molecules and proteins is a ubiquitous goal at the interface of biology and chemistry. The problem is approached from various different directions and with diverse purposes in mind. Much of this section will discuss the use of cheminformatics methods to identify likely interactions between ligands, as the organic molecules are collectively called, and proteins. Such predictions may have many uses in terms of understanding the likely bioactivities of molecules and both cellular and molecular functions of proteins.

The prediction of pharmaceutically relevant molecular properties has been the default problem addressed by cheminformatics throughout its history. The most obvious application, and a useful source of financial support, for cheminformatics research has been drug discovery. The label “drug discovery,” however, obscures the complexity of a variety of distinct questions. One objective, early in the drug discovery pipeline, is the identification of lead compounds, molecules possessing modest pharmacological activity that are starting points for chemical modifications enhancing their potency, selectivity and bioavailability. Subsequently, lead optimization will require Quantitative Structure-Activity Relationship (QSAR) studies to understand which modifications will best enhance affinity while, for instance, maximizing solubility and avoiding regions of chemical space likely to lead to toxicity.

**Figure 3:**
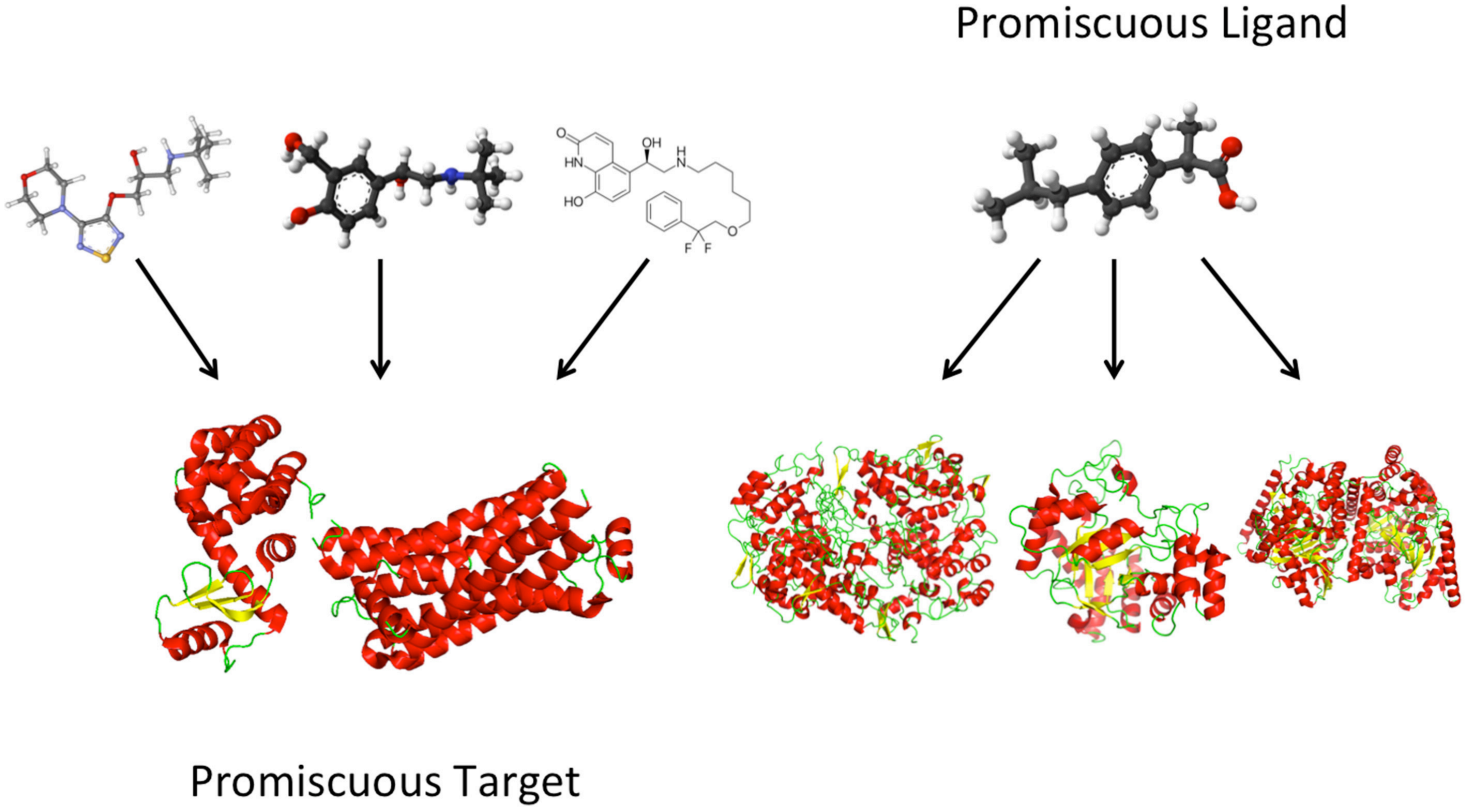
**Illustrations of the cases of a promiscuous ligand and a promiscuous target (left and right, respectively)**.

Protein target predictions (Bender et al., 2007; Lounkine et al., 2012) allow us to link molecular interactions to biological effects, and hence to identify and rationalize the bioactivities of compounds. Since many molecules interact promiscuously with several targets as well as different ligands interacting with the same target (Figure 3), we must predict off-target as well as on-target interactions. Protein-ligand interactions, other than those with the expected pharmacological protein target, can help to identify opportunities for drug repurposing (Kinnings et al., 2011; Napolitano et al., 2013), where a drug developed for one disease is able to treat a different condition. Such compounds have the advantage of already having been optimized for bioavailability and non-toxicity. More adventurously, polypharmacology (Chen et al., 2009) is possible, where deliberate use is made of the drug's ability to hit two targets, permitting more subtle modulation of its effect on a disease. Just as importantly, off-target prediction can identify likely side-effects (Lounkine et al., 2012) and adverse drug reactions (Bender et al., 2007).

### Similarity-based methods

One of the core methodologies of cheminformatics is the use of molecular similarity to predict bioactivity. In the simplest single-target cases, an *in silico* library of chemical structures is compared with those chemical structures known to possess bioactivity against that protein. Molecules are usually represented by one of the many sets of fingerprints or descriptors (Steinbeck et al., 2003; Bender et al., 2004) that distil various chemical, topological and physicochemical properties of a molecular structure into a string of tens to hundreds of bits. This exercise is predicated on the Similar Property Principle, that structures close together in the vector space defined by the descriptors should possess similar chemical or biochemical properties. Thus, proximity in an arbitrary chemical space is used as a proxy for likely similarity of properties. This is an extremely common approach for lead identification.

### Adaptation of similarity approaches to off-target prediction

We have created our own similarity-based procedure that has two significant modifications and is particularly suitable for use on multi-target problems with some missing data. This workflow has been applied in our work to two specific problems: the identification of performance-enhancing molecules that should be prohibited in sports (Mavridis and Mitchell, 2013), and predicting multi-target bioactivities of potential polypharmacological compounds for treatment of neurological diseases (Nikolic et al., 2015b).

The first methodological modification is that we do not base our search on single known actives, but rather on families of compounds. We define our families on the twin criteria of bioactivity against a particular protein family and cluster membership (Mavridis et al., 2013) of structurally similar ligands. For each target, we obtain one or often more *refined families*, as we call them, of compounds sharing both a structural scaffold and a target-specific bioactivity in common.

Our second modification was to devise a quantitative method of estimating the probability that a given query molecule is associated with a particular bioactivity-scaffold combination defining one specific *refined family*. This allows us to make comparable predictions across both on-target and off-target activities based on the current 1,715,667 compounds and 10,774 targets in the ChEMBL database (Gaulton et al., 2012). Doing this requires us to create a common scale for the different affinity measures reported in the literature, (IC50, Ki, Kd, EC50, ED50, potency, activity, inhibition) relevant to bioactivity. We applied a number of rules in order to generate sets of molecules experimentally reported to be bioactive, given affinities defined using the eight different measures, separating these from sets of inactive molecules. Those empirical rules were derived by considering the distributions of the different affinity measures amongst reported active and inactive compounds. Subsequently, we use the Parzen-Rosenblatt (PR) (Rosenblatt, 1956; Parzen, 1962) kernel density estimation method to transform Tanimoto similarities into probabilities of family membership.

**Figure 4:**
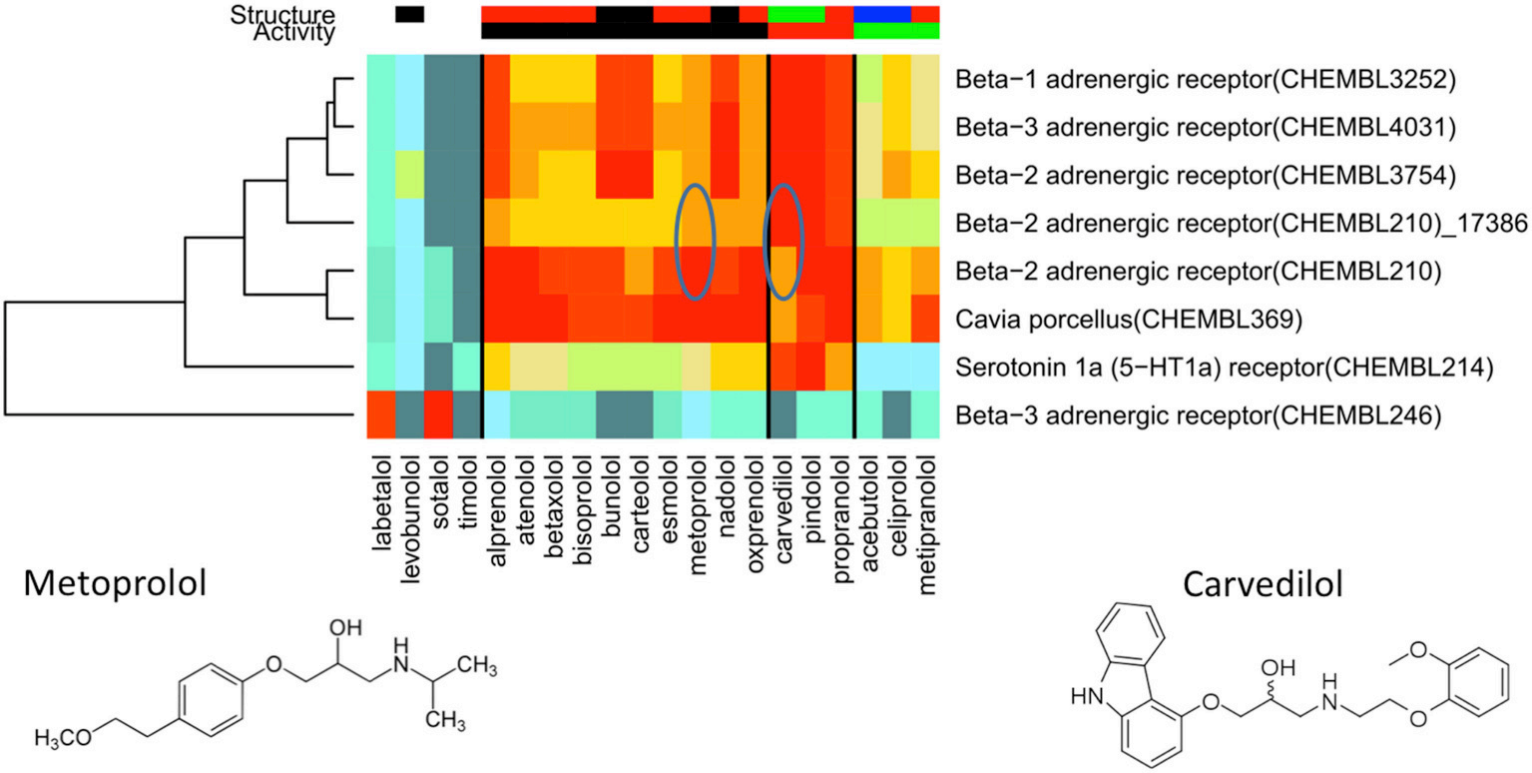
**Example case for the World Anti-Doping Agency data: the assignment of prohibited beta blockers to the Beta-2 adrenergic receptor family of ChEMBL (210)**.

Our study of molecules related to doping in sport (Steinbeck et al., 2003) used protein target prediction to predict athletic performance-enhancing properties. In it, we demonstrated that the freely available ChEMBL database can be clustered into bioactivity-based refined families of ligands, using our clustering algorithm PFClust (Mavridis and Mitchell, 2013). These refined families consist of distinct sets of compounds, each set with its own molecular scaffold. For example, we separated the ligands for the beta-2 adrenergic receptor, a target hit by many beta blockers, into two distinct families with each ligand generating a high probability of belonging to just one or other of the two groups and a lower score for the alternative refined family, as shown in Figure 4. The use of such structurally distinct refined families significantly improved our method's performance in cross-validation, to the extent of giving encouraging concordance with experiment. Overall, two thirds of our test cases in cross-validation had the correct refined family as the number one prediction, and seven eighths had this “correct” family among the top four hits. We sometimes found many different scaffolds for one target; for the androgen receptor ligands PFClust generated 126 different refined families. Even where we have no experimental data, we can still undertake predictions of the likely bioactivity. We identified the protein targets corresponding to seven of the World Anti-Doping Agency's defined prohibited classes of compounds; we found a mixture of expected and surprising protein targets. Many of the apparently unexpected targets, however, turned out to have published biochemically or clinically validated links to the relevant bioactivities.

We recently studied (Nikolic et al., 2015b) multi-target ligands intended to interact with MAO A and B; acetylcholinesterase (AChE) and butyrylcholinesterase (BuChE); or with histamine N-methyltransferase (HMT) and histamine H3-receptor (H_3_R). These enzymes are all potential drug targets for neurological conditions, including depression, Alzheimer's disease, obsessive disorders, and Parkinson's disease. Three groups of dual or multi-target compounds facilitated the generation of 3D-QSAR models for activity against the aforementioned protein targets. The first set of ligands consisted of novel carbonitrile–aminoheterocyclic compounds, designed to inhibit both MAO A and B. Amongst these, dicarbonitrile aminofuran derivatives were generally more selective MAO A inhibitors. The second group included acetylene-, indol-, piperidine- and pyridine-derivatives, which exhibited polypharmacology against MAO A/B, AChE, and BuChE. These agents are putative multitarget compounds against Alzheimer's disease. The third set of ligands contained multipotent histamine H3R antagonists that can concurrently inhibit HMT, and are therefore two-target procognitive compounds with potential therapeutic application against several psychiatric and neurodegenerative diseases. We used the Parzen–Rosenblatt kernel approach to build probabilistic models for both primary targets and off-targets, using data collected from the ChEMBL (Nikolic et al., 2015b) and DrugBank (Knox et al., 2011) databases. The cheminformatics-based target identifications agreed with four 3D-QSAR models for the various receptors, and with *in vitro* assays for serotonin 5-HT_1A_ and 5HT_2A_ receptor binding of the most promising ligand. As a result of this work, this and several other multi-target ligands were chosen for further investigation of their possible additional beneficial pharmacological activities.

Ain et al. (2014) used both protein and ligand descriptors to model the multi-target inhibitory profiles of serine proteinase inhibitors. They built separate sets of Random Forest (Breiman, 2001) models, some using only ligand descriptors, which they called “QSAR models,” and others built using also protein descriptors, namely “proteochemometric (PCM) models”. Across 12,625 inhibitors and 67 targets, they found that the models including protein descriptors performed substantially better than ligand-only ones, in terms of both R^2^ (0.64 v 0.35) and root mean squared error (0.66 v 1.05 log units). They found that both the binding site amino acids and the protein sequence corresponded to important protein descriptors in their models.

### Relationship to protein structure prediction

Ain et al.'s finding that the best models require protein information (Ain et al., 2014) is particularly interesting. We recently asked *why* sequence-based protein function prediction methods work so effectively. For example, De Ferrari et al. obtained 98% prediction accuracy for enzyme function, based on transferring annotations from a query sequence's nearest neighbor of known function (De Ferrari et al., 2012). We have recently demonstrated that the majority of the predictive power of such sequence signature-based methods comes from the wealth of evolutionary information contained in the whole sequence, and only a small part of the predictive ability emanates from the many fewer functionally essential conserved residues (Beattie et al., 2015). In the context of polypharmacology and off-target interactions, structure-based methods of protein function prediction (Laskowski et al., 2005; Pal and Eisenberg, 2005; Cuff et al., 2011) are also highly relevant. Unexpected ligand-target interactions can be discovered by cross-docking the library of compounds into the active sites of the known structures of the various proteins (Favia et al., 2008; Patel et al., 2015). This methodology, however, requires an experimental protein structure, or at least a high-quality structural model, for the target, and is computationally much more expensive than cheminformatics.

As well as predicting interactions with a protein's major functional site, which we term orthosteric, it is also possible to predict allosteric ligand function. For example, van Westen et al. predicted allosteric behavior of compounds based on structural and chemical descriptors and data from ChEMBL (van Westen et al., 2014). As well as such predictions of allosteric molecules, it is also important to be able to predict which proteins will be amendable to allosteric proteins and which residues or clefts may be involved. We have recently used a Random Forest model to predict the presence of allosteric binding sites on proteins, based on structure, solvent accessibility and predicted binding affinity (Chen et al., 2016). Other predictions of allostery are derived from reduced models of protein dynamics, for instance using normal mode analysis (Panjkovich and Daura, 2012) or modeling energy flow within the protein structure (Erman, 2011).

## Virtual screening of multitarget compounds for CNS diseases

**Figure 5:**
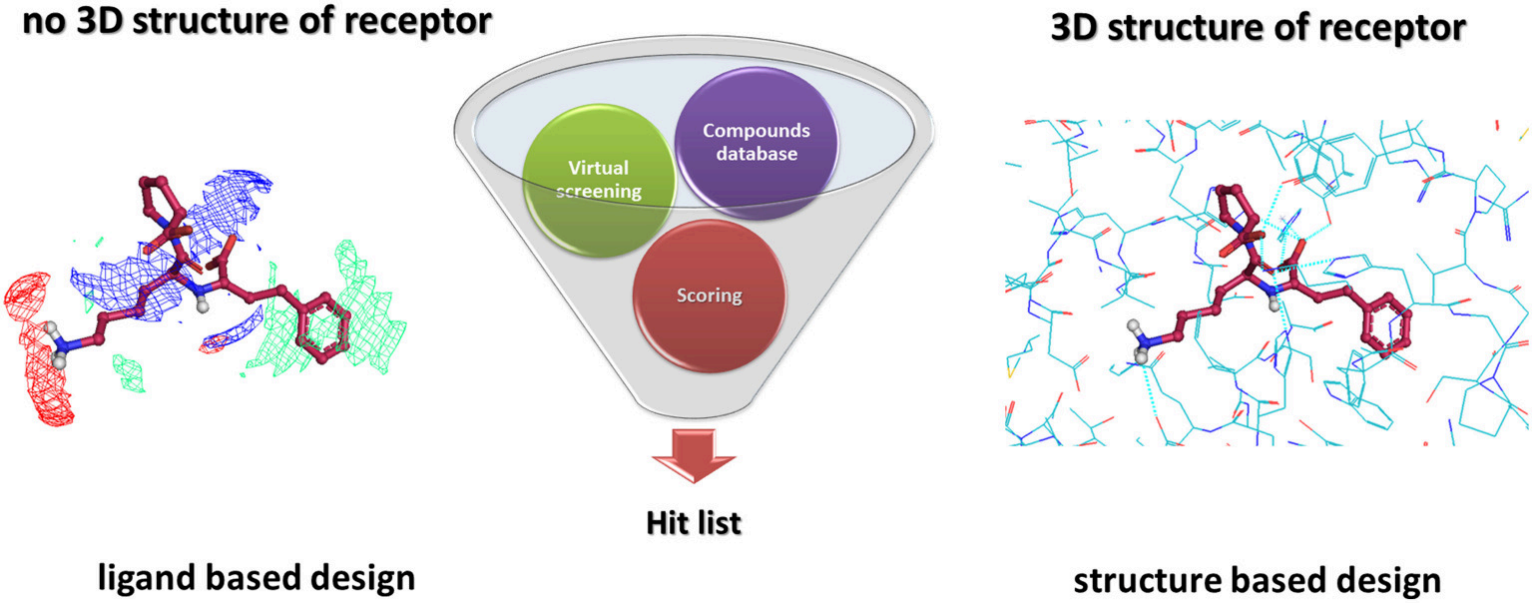
**Schematic representation of the virtual screening strategy**.

Virtual Screening (VS) is widely used in drug discovery to reduce the enormous compound collections to a more manageable number for further synthesis and biological *in vitro* testing (Alvarez, 2004). The application of computational technologies has allowed medicinal chemists to develop new drugs in a time and cost-effective manner. Two generally accepted VS methods used in Computer Aided Drug Design (CADD) are classified as Ligand-Based Virtual Screening (LB-VS) and Structure-Based Virtual Screening (SB-VS) (Figure 5; Wilson and Lill, 2011). Ligand-based VS approaches are often applied when no structural information about the target protein is available and analyze the physicochemical similarity between large compound databases and known active molecules. On the other hand, the structure-based VS approach applies different modeling techniques, often including docking, to mimic the binding interaction of ligands to a biomolecular target. In both VS approaches, structures from virtual libraries or commercial databases are compared to the template and scored. In recent years, besides the individual application of ligand- and structure-based VS methods, combined techniques have also been proposed (Sperandio et al., 2008; Wilson and Lill, 2011). Even though docking is the most widely used approach in early phase drug discovery, a recent study has shown that ligand-based virtual screening methods in general yield a higher fraction of potent hits (Ripphausen et al., 2010). It is also important to note that hits with low nanomolar potency are rarely identified by VS (Eckert and Bajorath, 2007). However, compounds for further chemical exploration are predominantly provided.

Ligand-based VS applies two-dimensional (e.g., 2D-fingerprint) or three-dimensional (e.g., 3D-pharmacophore) searches between large compound databases and already known active molecules. This technique essentially neglects the target structure and allows a prioritization of molecules based on the Similar Property Principle, the assumption that compounds with similar descriptors tend to exhibit similar biological activity (Koeppen et al., 2011). Typically, topology-based descriptors of the known active compounds and the potential bioactive hits are compared to quantify molecular similarity. A major problem related to similarity methods is their bias toward input molecules and difficulty in making decision which structure to use as input (Venkatraman et al., 2010).

Beside similarity searching, the ligand-based pharmacophore method is also applied in VS. Pharmacophore models are usually built by using a set of structurally and functionally diverse ligands. This method is not only used to identify novel hit compounds, but also for profiling and anti-target modeling to avoid side-effects resulting from off-target activity (Schuster, 2010). However, ligand-based virtual screening is often applied in combination with structure-based approaches to identify potential hit molecules.

In contrast to ligand-based approaches, which allow the identification of chemically similar ligands, SBVS offers the possibility of discovering ligands with new scaffolds or chemical functional groups. SBVS categories, such as shape similarity, structure-based pharmacophores and docking, require knowledge of the three-dimensional structure of target protein. Structures for target proteins are usually obtained by X-ray crystallography or nuclear magnetic resonance (NMR) spectroscopy. In cases when this information does not exist, which is common in membrane receptors such as GPCRs, homology models can be used instead (Cavasotto, 2011). Structure-based pharmacophore models are developed on the basis of the active site and can be used to screen a compound database. Such pharmacophores are obtained by investigating all possible interaction sites in a binding pocket (Leach et al., 2010). Energy-based and geometry-based methods are applied to identify potentially important interaction sites and translate them into pharmacophore features. Typically, a binding pocket has a higher number of potential interaction sites than are normally observed actually being used in protein-ligand complexes.

**Figure 6:**
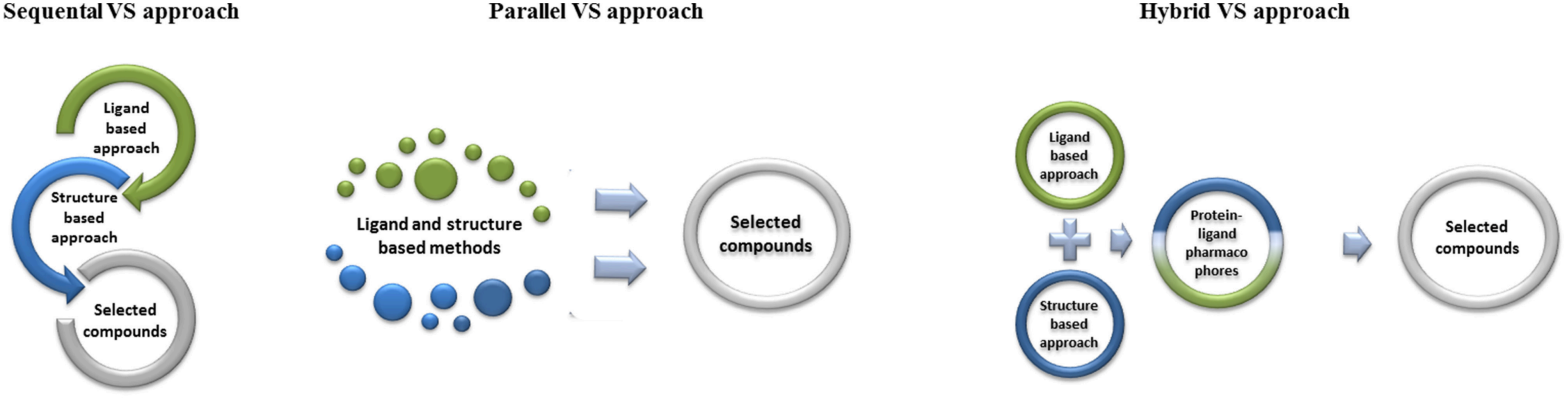
**Sequential, parallel or hybrid combinations of VS techniques**.

The combination of the structure- and ligand-based VS strategies also occurs in many CADD studies (Drwal and Griffith, 2013). Sequential, parallel or hybrid combinations of VS techniques take into account all available chemical and biological information and thereby mitigate the drawbacks of each individual method (Figure 6; Hein et al., 2010; Wilson and Lill, 2011). Most of the recently published CADD studies apply a *sequential* VS approach (Khan et al., 2010; Weidlich et al., 2010; Drwal et al., 2011; Banoglu et al., 2012). In this approach ligand- and structure-based strategies are used in the VS protocol to gradually filter the large databases until the number of remaining potential hit compounds is small enough for biological testing. In *parallel combination* of VS methods, ligand- and structure-based strategies are run independently. Top ranked hit compounds are selected by a consensus aproach and processed for further biological testing. Benchmarking studies with retrospective analysis of performance have shown that successful application of parallel methods in VS is possible (Tan et al., 2008; Swann et al., 2011; Svensson et al., 2012). The *Hybrid VS approach* integrates ligand- and structure-based methods into one technique (protein-ligand pharmacophores) in order to enhance the accuracy of performance (Chen et al., 2010; Postigo et al., 2010; Spitzer et al., 2010; Drwal et al., 2011; Planesas et al., 2011).

During the last decade, many virtual screening methods have been developed and applied to discover novel potent ligands for the treatment of various neurological diseases. Several successful cases of reported virtual screening studies used to identify promising hits for CNS drugs discovery have been listed in Table 5. In this chapter we provide an overview of some of them that are significant from the polypharmacological point of view.

**Table 5:** **Reported virtual screening studies used in CNS drug discovery**.

**Compounds**	**CNS diseases**	**Virtual screening method**	**Software package**	**References**
BACE1 (beta-secretase 1) inhibitors	AD	SB approach based on pharmacophore model and molecular docking	LigandScout 1.03 *www.inteligand.com* GOLD 3.2 *www.ccdc.cam.ac.uk*	Vijayan et al., 2009
NK3 receptor antagonists	Schizophrenia; depression; anxiety	Sequential similarity analysis followed by CoMFA	ROCS 2.4.1. and 3.0 *www.eyesopen.com*	Geldenhuys et al., 2010
AChE inhibitors	AD	LB approach based on pharmacophore model and molecular docking	Discovery Studio 2.5.5 *www.accelrys.com* LibDock (Diller and Merz, 2001)	Lu et al., 2011
Human DOPA Decarboxylase Inhibitors	PD	SB approach based on pharmacophore model and molecular docking	MOE; Dovis 2.0; (Jiang et al., 2008) AutoDock Vina *http://vina.scripps.edu/*	Daidone et al., 2012
Histamine H_3_receptor ligands	PD; AD; epilepsy; sleeping disorders	LB and structure-based virtual fragment screening	FLAP *www.moldiscovery.com*	Sirci et al., 2012
MAO-B inhibitors	PD	LB virtual screening based on scaffold hopping approach	vROCS 3.0 *www.eyesopen.com*	Geldenhuys et al., 2012
SERT (serotonin transporter) Inhibitors	Depression	LB virtual screening based on two- and three-dimensional similarities; flexibile docking	JChem *www.chemaxon.com* Discovery studio	Gabrielsen et al., 2014
BuChE inhibitors	AD	LB virtual screening based on two- and three-dimensional similarities	LiSiCA	Lešnik et al., 2015
Serotonine 5-HT_6_ antagonists	AD; schizophrenia; obesity	LB virtual approach based on two-dimensional similarities and pharmacophore model	InstJChem; JChemForExcel; *www.chemaxon.com* Phase-program *www.schrodinger.com*	Dobi et al., 2015
H_3_R antagonist/5HT_4_R agonist	AD	LB approach based on pharmacophore model similarity based clustering method and molecular docking	Discovery Studio 3.5 *www.accelrys.com* LibMCS http://www.chemaxon.com/jchem/doc/user/LibMCS.html Glide Induced-Fit Docking http://www.schrodinger.com/Induced-Fit/	Lepailleur et al., 2014
BACE-1/GSK-3 β activity	AD	SB approach based on molecular docking followed by Tanimoto ligand similarity	Monte Carlo stochastic optimizer implemented in ICM (Abagyan and Totrov, 1994)	Bottegoni et al., 2015

Lepailleur and co-workers applied pharmacophore-based virtual screening in combination with similarity based clustering method and molecular docking to identify dual H_3_R antagonist/5HT_4_R agonists (Lepailleur et al., 2014). Novel ligands would have potential for treatment of neurodegenerative diseases such as Alzheimer's disease. A three-dimensional pharmacophore model was constructed based on a set of six H3R antagonists developed by different pharmaceutical companies, using Catalyst software implemented in Discovery Studio 3.5 (Accelrys Inc., San Diego, CA, USA). This model was used as a search query for virtual screening of the CERMN chemical library (*www.cermn.unicaen.fr*) with a focus on serotonin (5-HT) “privileged structures“. Binding experiments confirmed that benzo[h]-[1,6]naphthyridine ligands selected by this VS approach exert high affinity for both H3 and 5-HT_4_ receptors. Recently, Bottegoni et al. carried out a virtual ligand screening protocol to identify fragments that display considerable activity at both β-secretase 1 (BACE-1) and glycogen synthase kinase 3β (GSK-3β) (Bottegoni et al., 2015). Discovery of multitarget drugs which are able to modulate BACE-1 and GSK-3 β activity simultaneously represents a promising strategy in the treatment of Alzheimer's disease. In this study, a VS approach based on docking simulations and Tanimoto similarity analysis was applied on the ZINC database (*www.zinc.docking.org*). Top ranked compounds selected by VS were tested *in vitro* and one with activitiy in the low-micromolar range at both enzimes was identified as a hit. Potential acetylcholinesterase inhibitors were also discovered using a virtual screening approach, in combination with molecular docking (Lu et al., 2011). Three-dimensional pharmacophore models were constructed based on a set of known AChE inhibitors. Virtual screening performed on the National Cancer Institute (NCI) compound database obtained nine new inhibitors that can block both catalytic and peripheral anionic sites of AChE. Designing or identifying dual-acting inhibitors that block both AChE binding sites is essential in preventing the degradation of acetylcholine in the brain and in protection of neurons from Abeta (Aβ) toxicity.

Finally it can be concluded that most of the recent successfully performed drug discovery studies used a sequential combination of ligand and structure-based virtual screening techniques, with particular focus on pharmacophore models and the docking approach.

### Docking of multi-target compounds for neurodegenerative diseases

Docking is a computational technique that predicts the preferred orientation of one molecule toward the other (Lengauer and Rarey, 1996). It is widely utilized as a hit identification and lead optimization tool, before compound synthesis, if the structure of the target is reliably known (Kitchen et al., 2004). In this chapter, the focus will be on some of the most commonly used docking software (Table 6).

**Table 6:** **Recent docking studies employed to identify potential inhibitors for neurological targets**.

**Compounds**	**CNS diseases**	**Software package**	**References**
MAO-A inhibitors	Depression	AutoDock http://autodock.scripps.edu/	Evranos-Aksoz et al., 2015
Metallothionein-III inhibitors	AD	Discovery Studio 2.5.5 *www.accelrys.com*	Roy et al., 2015
Sirtuin inhibitors	AD	GLIDE http://www.schrodinger.com/Glide	Karaman and Sippl, 2015
MAO-A, MAO-B, AChE, BuChE inhibitors	AD	GLIDE http://www.schrodinger.com/Glide	Bautista-Aguilera et al., 2014b,c
AMPK2 inhibitors	Stroke	AutoDock, FlexX http://autodock.scripps.edu/ https://www.biosolveit.de/FlexX/	Park et al., 2014
MAO- B inhibitors	PD, AD	AutoDock, GOLD, LibDock	Yelekci et al., 2013
Dopamine transporter inhibitors	ADHD, PD, depression and addiction	MOE https://www.chemcomp.com/	Schmitt et al., 2010

Ligand-protein docking samples the conformations of small molecules—igands—in binding sites of proteins, and scoring functions are used to evaluate which of these conformations best fits the protein binding site (Warren et al., 2006). Thus, it calculates and ranks the complexes resulting from the association between a certain ligand and a target protein of known three-dimensional structure (Sousa et al., 2006). Initially rigid docking was used, where both target and compound were rigid. However, advances in both software and computer power mean that full flexibility on the ligand can now be allowed, and this approach is the most popular now. There are three general kinds of algorithms formulated to apply ligand flexibility: systematic methods, random or stochastic methods, and simulation methods. Systematic search algorithms explore all the degrees of freedom in a molecule, random search algorithms explore the conformational space by applying random changes to a single ligand or a group of ligands, and simulation methods utilize a different approach to the docking process such as molecular dynamics (MD) or energy minimization methods (Sousa et al., 2006). Also, many scoring functions have been reported over the years, and classified as force-field-based, empirical, and knowledge-based. The first category uses available force fields to calculate the direct interactions between protein and ligand atoms (frequently comprising the non-covalent energy terms covering the electrostatic energy, the van der Waals (vdW), and hydrogen bonding). Secondly, an empirical scoring function calculates the fitness of protein—ligand binding by summing up the contributions of various individual terms, each representing a significant energetic factor in protein—ligand binding. The third type of method utilizes pairwise statistical potentials between protein and ligand, based on the occurrence frequencies of particular atom-atom interaction frequencies in databases of protein-ligand complex structures (Mitchell et al., 1999). Recently, methods that bring pharmacophore and structure—activity relationship (SAR) analysis into protein—ligand interaction assessment have been introduced, representing new trends in this field (Hu and Lill, 2014).

Today, there are numerous docking software packages available, based on different search algorithms and scoring functions. None of the existing docking programs and scoring functions is uniquely excellent and the improvements are still continuing. The best way is to apply several docking programs in order to reduce the artifacts. The three widely used software tools are CDOCKER (Wu et al., 2003), GOLD (Jones et al., 1995) and AutoDock (Morris et al., 1998, 2009).

**Figure 7:**
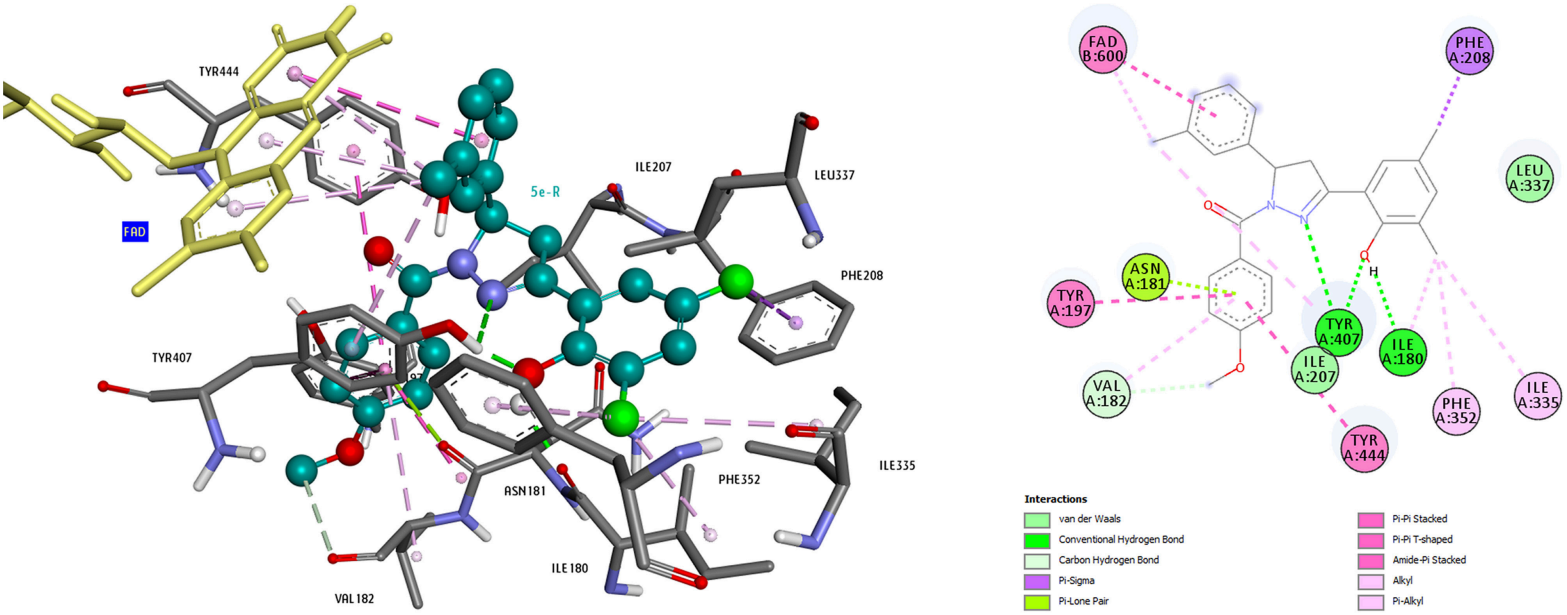
**3D and 2D representations of compound 5e(R) binding mode in the active site of MAO-A**.

**Figure 8:**
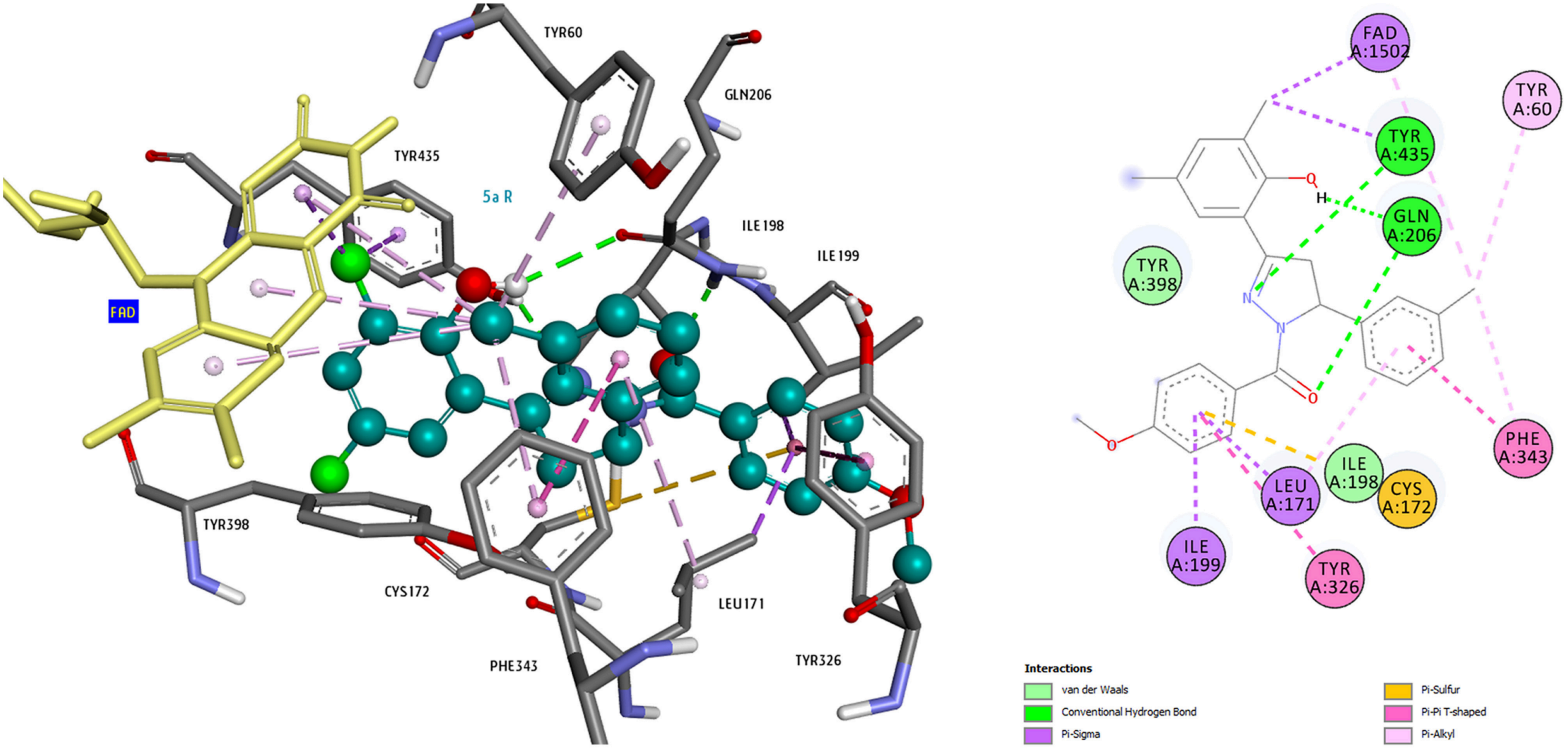
**3D and 2D representations of compound 5e(R) binding mode in the active site of MAO-B**.

The usage of docking tools in discovery of novel compounds for neurodegenerative diseases could be explained through the example of our study on MAO -A and B inhibitors. The crystal structure of human MAO-A (hMAO-A) complexed with the reversible inhibitor harmine (PDB 2Z5X) (Son et al., 2008) and the crystal structure of human MAO-B (hMAO-B) co-crystallized with the reversible inhibitor safinamide (PDB 2V5Z) (Binda et al., 2007) were extracted from the Protein Data Bank (PDB) (Berman et al., 2000; http://www.rcsb.org) for protein setup. Studies were carried out on only one subunit of the enzymes. Each structure was cleaned of all water molecules and inhibitors and all non-interacting ions were removed before being used in the docking studies. For each protein, all hydrogens were added and the protein is minimized using the Discovery Studio protocol (accelrys.com), assigning a CHARMM force field. Missing hydrogen atoms were added on the basis of the protonation state of the titratable residues at a pH of 7.4. Ionic strength was set to 0.145 and the dielectric constant was set to 10. Molecular models of the inhibitors were built and optimized using SPARTAN 10.0. Docking was performed using AutoDock 4.2. For coordinates of the binding pocket, the N5 atom of the FAD molecule was taken, and the chosen region covers the entire binding site and its neighboring residues. Compounds were docked in both MAO-A and MAO-B and the selectivity was compared. To study the binding pose of these compounds, several representative ligands were chosen, the important interactions were visualized in the Accelrys Visualization 4.5. program. Analysis of binding modes revealed that aromatic groups of these compounds in hydrophobic cage of MAO-A and MAO-B enzymes were important for affinity (Figures 7, 8; Evranos-Aksoz et al., 2015).

## Concluding remarks

Extensive use of computational methods such as data mining, cheminformatics, QSAR modeling, virtual screening and docking, provide a time and cost efficient drug discovery processes.

These methods have become an integral part of drug discovery. A wide range of computational tools is being developed and used to obtain hits that are more likely to give potential clinical candidates. However, despite their success, both ligand- and structure-based techniques face challenges and limitations that should be considered during application. In recent years, integration of various cheminformatic, QSAR, virtual screening and docking protocols has become very popular, since it enhances their strength and applicability. This chapter focuses on various computation methodologies successfully applied in CNS drug discovery processes, such as design of novel donepezil–indolyl hybrids, N-Methyl-N-((1-methyl-5-(3-(1-(2-methylbenzyl)piperidin-4-yl) propoxy)-1H-indol-2-yl)methyl)prop-2-yn-1-amine, and donepezil-pyridyl hybrids, as multitarget inhibitors of acetylcholine esterase and MAO (AChE/BuChE/MAO-A/MAO-B) that were effective drug candidates for therapy of neurodegenerative Alzheimer's (AD) and Parkinson's diseases (PD).

Detailed analysis of the recently reported case studies revealed that the majority of them use a sequential combination of ligand and structure-based virtual screening techniques, with particular focus on pharmacophore models and the docking approach.

## Author contributions

All authors contributed to the conception and interpretation of the work and to its critical revision. All authors have approved the final version and may be held accountable for the integrity of this review of current literature.

### Conflict of interest statement

The authors declare that the research was conducted in the absence of any commercial or financial relationships that could be construed as a potential conflict of interest. The handling Editor declared a shared affiliation with one of the authors [JM] and states that the process nevertheless met the standards of a fair and objective review.

## Abbreviations

MTDL: multi-target designed ligands
QSAR: quantitative structure-activity relationship
AD: Alzheimer's disease
PD: Parkinson's disease
AChE: acetylcholinesterase
BuChE: butyrylcholinesterase
MAO: monoamine oxidase
Aβ: amyloid beta
5-HT: serotonin receptor
D-R: dopamine receptor
H-R: histamine receptor
GPCRs: G protein–coupled receptors
HMT: histamine N-methyltransferase
SERT: serotonin transporter
AMPK: 5′'-adenosine monophosphate-activated protein kinase.
